# Fluorinated and Bio-Based Polyamides with High Transparencies and Low Yellowness Index

**DOI:** 10.3390/polym10121311

**Published:** 2018-11-27

**Authors:** Kenji Takada, Yuko Mae, Tatsuo Kaneko

**Affiliations:** 1 Graduate School of Advanced Science and Technology, Energy and Environment Area, Japan Advanced Institute of Science and Technology, 1-1 Asahidai, Nomi, Ishikawa 923-1292, Japan; takada@jaist.ac.jp (K.T.); s1610172@jaist.ac.jp (Y.M.); 2 Japan Science and Technology, JST ALCA, Tokyo 102-0076, Japan

**Keywords:** bio-based polymer, polyamides, fluorinated polymer, transparent materials

## Abstract

Bio-based polyamides with high transparency and low yellowness were synthesized using 4,4′-bis(trifluoroacetamido)-*α*-truxillic acid (ATA-F1) and 4,4′-bis(pentafluoropropionamido)-*α*-truxillic acid (ATA-F2) as a fluoroalkylated aromatic dicarboxylic acid, and various aromatic diamines. The introduction of fluorine side chains improved the transparency of the polyamide film, and suppressed its yellowness. On the other hand, water repellency, which should be a general characteristic of the fluorinated polymers, was not observed. By using ATA-F1 and various aromatic diamines, aromatic and fluorinated polyamides were obtained. In addition, these also demonstrated a high transparency and a low yellowness index. The heat resistance properties of all the obtained polyamides was over 250 °C, and the characteristics of the bio-based polyamides from 4-aminocinnamic acid derivatives were retained.

## 1. Introduction

Synthesizing and enhancing bio-based plastics can contribute not only to the promotion of the use of renewable resources, but also to the reduction of atmospheric carbon dioxide. Bio-based plastics with high transparencies have become widespread in recent decades, and they include polylactide, which is derived from biomolecules [1,2]. In addition, polylactide has additional characteristics, such as biodegradability and biocompatibility, and it could be used as a soft bio-based plastic. On the other hand, there are only a few examples of bio-based plastics that have high heat resistances of over 150 °C, and high strength properties, such as engineering plastics. Furthermore, when focusing on transparency, there are even fewer examples. As a limited example, we have developed polyimide [3,4], polyamide [5], and polyurea [6], using 4-aminocinnamic acid produced from glucose by microorganisms. The abovementioned bio-based plastics have high thermal resistivities far exceeding the value of generally used transparent plastics that have a thermal decomposed temperature of over 150 °C. In particular, the 4-aminocinnamic acid-derived polyamide (PA) was reported to be bio-derived, with high transparency and mechanical properties, and it was expected to be developed as an alternative material to glass. However, it was difficult to completely suppress the yellow coloring of the molded film. The PA contained aromatic rings in the main-chain, and thus, it caused the yellowing of the molded film. The introduction of fluorine atoms is the one of the most effective methods for suppressing the yellowing of the film or resin. In the case of aromatic polymers, most of them are colored yellow or brown, due to intra- and intermolecular charge transfer (CT) interactions [7]. Using a fluorine-containing monomer is typically employed as a means for improving the light transmittance in the visible light region. The transparency of a polymer film can be improved by this approach. For instance, Matsuura and coworkers developed a highly transparent polyimide synthesized from 2,2′-bis(trifluoromethyl)-4,4′-diaminobiphenyl anhydride and 4,4′-(hexafluoroisopropylidene)diphthalic anhydride [8]. Ando and coworkers synthesized perfluorinated polyimide from perfluoroaromatic diamine and perfluorinated dianhydride, 1,4-bis(3,4-dicarboxytrifluorophenoxy)tetrafluorobenzene dianhydride, which presented the possibility of a transparent resin [9]. From these examples, the difference in charges between the monomer units was reduced by the introduction of fluorine, and the CT was suppressed by sparsely packing the molecular chains with bulky fluoroalkyl groups. Although these examples were related to aromatic polyimides, bio-polyamides also have aromatic rings in the main-chain structure, and therefore, an improvement in transparency could also be expected by using the same method. By applying the molecular design of these transparent resins to bio-polyamide, it is possible to increase the transparency and to suppress the yellowing of the obtained resin. In this study, fluorinated polyamide was synthesized for improving the transparency and performance of the bio-based plastics, by investigating the film-preparing conditions and evaluating its transparency.

## 2. Materials and Methods

### 2.1. Materials

4-Aminocinnamic acid, pentafluoropropionic anhydride, triphenyl phosphite, 4,4′-diaminodiphenyl ether, 4,4′-diaminodiphenylmethane, 2,2-bis(4-aminophenyl)hexafluoropropane, 4,4′-diaminobenzophenone, and 4,4′-diaminodiphenyl sulfone were purchased from Tokyo Chemical Industry Co., LTD, Tokyo, Japan. Trifluoroacetic anhydride, triethylamine, and *N,N*-dimethylacetamide were purchased from FUJIFILM Wako Pure Chemical Corporation. Dichloromethane, methanol, ethanol, pyridine, *N,N*-dimethylformamide (DMF), and *N*-methyl-2-pyrrolidone (NMP) were purchased from Kanto Chemical Co., Inc, Tokyo, Japan. All chemicals were directly used as purchased. 4,4′-Diamino-*α*-truxillic acid and 4,4′-diamino-*α*-truxillic acid dimethyl ester were synthesized based on previous research [4,5,10].

### 2.2. Instruments

Proton (^1^H) nuclear magnetic resonance (NMR) and carbon-13 (^13^C) NMR measurements were carried out using Bruker Bio-spin AG 400 MHz and 100 MHz, respectively. Gel permeation chromatography (GPC) was performed on the Shodex column (SB 806M × 2), column oven (GL Science Tokyo, Japan, CO 631A, set as 40 °C), degassing unit (GL Science, DG 660B), pump (JASCO, Tokyo, Japan, PU-2080 Plus), refractive index detector (JASCO, 830-RI), and ultraviolet detector (JASCO, UV-2075 Plus) using 0.01 mol L^−1^ of lithium bromide (LiBr) solution of DMF as an eluent (flow rate, 1 mL min^−1^). Electrospray ionization Fourier transform ion cyclotron resonance mass spectrometry (ESI-FT-ICR MS) was performed by using Bruker Solarix-JA. Thermal gravimetrical analysis (TGA) was carried out by using a HITACHI STA 7200 under nitrogen flow (flow rate, 200 mL min^−1^) from 25 to 800 °C with a heating rate of 5 °C min^−1^ to determine the 5% and 10% decomposition temperature (*T*_d5_ and *T*_d10_). Differential scanning calorimetry (DSC) was performed by SEIKO X-DSC7000T to measure the glass transition temperature (*T*_g_), which were measured from 25 to 300 °C at a heating rate of 10 °C min^−1^ with 5 mg (ca.) of sample. Ultraviolet-visible (UV-Vis) absorption spectroscopy was performed by V-670 (JASCO) from 200 to 800 nm as the measured range. The contact angle was measured using a Drop-Master DM 300.

### 2.3. Synthesis of 4-(Trifluoroacetamido)cinnamic Acid

4-Aminocinnamic acid (2.50 g, 15.3 mmol) was dissolved in a triethylamine (2.76 mL, 19.9 mmol) and dichloromethane (30 mL) solution. Trifluoroacetic anhydride (2.81 mL, 19.9 mmol) was then added to the reaction mixture at 0 °C, which was subsequently warmed to room temperature and allowed to react for 12 hr. After the reaction, inorganic salt was removed via filtration, and the filtrated liquid was evaporated. Then, the obtained crude product was dissolved in methanol and distilled water with a volume ratio of 1/1, and the mixture was cooled to 0 °C for recrystallization to produce 4-(trifluoroacetamido)cinnamic acid as a yellowish powder with a yield of 3.41 g (95.2%). ^1^H NMR (400 MHz, DMSO-*d*_6_): δ (ppm) 6.50 (d, 1H, *J* = 16.0 Hz, –CH=C*H*–COOH), 7.56 (d, 1H, *J* = 16.0 Hz, –C*H*=CH–COOH), 7.73 (s, 4H, aromatic), 11.41 (s, 1H, –N*H*–), 12.40 (s, 1H, –COO*H*). ^13^C NMR (100 MHz): δ 114.3, 117.1, 119.0, 121.0, 129.1, 131.4, 138.0, 143.1, 154.6, 167.6. ^1^H and ^13^C NMR spectra were shown in Figures S1 and S2, respectively.

### 2.4. Synthesis of 4,4′-Bis(trifluoroacetamido)-α-Truxillic Acid (ATA-F1)

4-(Trifluoroacetamido)cinnamic acid (1.50 g, 5.79 mmol) was suspended in an *n*-hexane solution (50 mL), and ultraviolet (UV) light was irradiated using a high-pressure mercury (Hg) lamp. After UV irradiation for 10 hr, the obtained product was filtrated to provide 4,4′-bis(trifluoroacetamido)-*α*-truxillic acid as a white powder with a yield of 0.77 g (51.3%). ^1^H NMR (400 MHz, DMSO-*d*_6_): δ (ppm) 3.80 (dd, 2H, *J* = 7.2, 10.3 Hz, –C*H*–COOH), 4.28 (dd, 2H, *J* = 7.2, 10.2 Hz, –C*H*–C_6_H_4_–), 7.39 (d, 4H, *J* = 8.6 Hz, aromatic), 7.61 (d, 4H, *J* = 8.6 Hz, aromatic), 11.24 (s, 2H, –N*H*–), 12.14 (s, 2H, –COO*H*). ^13^C NMR (100 MHz): δ 40.5, 46.2, 114.4, 117.3, 120.8, 128.3, 134.9, 136.8, 154.4, 172.8. ^1^H and ^13^C NMR spectra and ESI-FT-ICR MS spectrum were shown in Figures S3, S4 and S7, respectively.

### 2.5. Synthesis of 4,4′-Bis(pentafluoropropionamido)-α-Truxillic Acid (ATA-F2)

Pentafluoropropionic anhydride (0.67 mL, 3.39 mmol) was added to a *N,N*-dimethylacetamide (25 mL) solution of 4,4′-diamino-*α*-truxillic acid (0.50 g, 1.53 mmol) at room temperature. After reacting for 3 hr, *N,N*-dimethylacetamide was removed by evaporation and distilled water (20 mL) was added to the resulting mixture. The obtained product was collected via filtration and recrystallized in ethanol to produce 4,4′-bis(pentafluoropropionamido)-*α*-truxillic acid as a white powder with a yield of 0.36 g (38.0%). ^1^H NMR (400 MHz, DMSO-*d*_6_): δ (ppm) 3.80 (dd, 2H, *J* = 7.2 Hz, 10.3 Hz, –C*H*–COOH), 4.28 (dd, 2H, *J* = 7.2 Hz, 10.3 Hz, –C*H*–C_6_H_4_–), 7.40 (d, 4H, *J* = 8.6 Hz, aromatic), 7.62 (d, 4H, *J* = 8.6 Hz, aromatic), 11.30 (*s*, 2H, –N*H*–), 12.15 (*s* 2H, –COO*H*). ^13^C NMR (100 MHz): δ 40.9, 46.5, 103.7–110.1, 113.5–122.8, 121.4, 128.5, 135.1, 137.4, 155.5, 173.2. ^1^H and ^13^C NMR spectra and ESI-FT-ICR MS spectrum were shown in Figures S5, S6 and S8, respectively.

### 2.6. Synthesis of Bio-Based Polyamide Using ATA-F1, ATA-F2

The experimental procedure was as follows: 4,4′-Diamino-*α*-truxillic acid dimethyl ester (49.0 mg, 0.14 mmol), ATA-F1 (72.5 mg, 0.14 mmol), and pyridine (0.06 mL, 0.87 mmol) were dissolved in *N*-methyl-2-pyrrolidone (0.15 mL). Triphenyl phosphite (0.04 mL, 0.15 mmol) was added to the reaction mixture, and the temperature was increased to 100 °C. After 3 h, the reaction mixture was reprecipitated in methanol and dried under reduced pressure to gain a quantitative yield of trifluoromethylated bio-based polyamide (PA-F1). PA-F2 was synthesized by using ATA-F2 (50.1 mg, 0.14 mmol), instead of ATA-F1, with the same procedure. PA-F1**a**, PA-F1**b**, PA-F1**c**, PA-F1**d**, and PA-F1**e** were synthesized using the abovementioned procedure with ATA-F1, and 4,4′-diaminodiphenyl ether (79.3 mg, 0.40 mmol), 4,4′-diaminodiphenylmethane (20.1 mg, 0.10 mmol), 2,2-bis(4-aminophenyl)hexafluoropropane (66.5 mg, 0.20 mmol), 4,4′-diaminobenzophenone (42.5 mg, 0.20 mmol), and 4,4′-diaminodiphenyl sulfone (24.5 mg, 0.100 mmol), respectively. For the preparation of polyamide film, the obtained polymers (10 mg) were dissolved in *N,N*-dimethylformamide (1.0 mL), and the polymer solution was cast into quartz glass. Only PA-F1 and PA-F2 could be peeled off, but these films had brittle properties. Thus, we could not measure their mechanical properties.

## 3. Results and Discussion

### 3.1. Synthesis and Structural Analysis of Fluorinated Bio-Based Polyamides (PA-F1 and PA-F2)

**Scheme 1:**
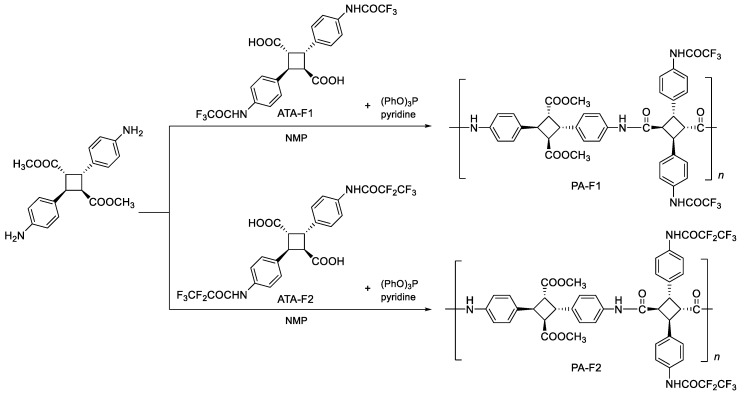
Synthesis of fluorinated polyamide, PA-F1 and PA-F2, using bio-based diamine and fluorinated dicarboxylic acid, ATA-F1 and ATA-F2, in the presence of triphenyl phosphite ((PhO)_3_ P) and pyridine in *N*-methyl-2-pyrrolidone (NMP).

In order to synthesize fluorinated bio-based polyamides, fluorinated dicarboxylic acid monomers of ATA-F1 and ATA-F2 were synthesized from 4-aminocinnamic acid as partially bio-based monomers. ATA-F1 was synthesized by the photodimerization of 4-(trifluoroacetamido)cinnamic acid in a heterogeneous Hg arc lamp (>250 nm) irradiation system. ATA-F2 was synthesized from 4-aminocinnamic acid dimer, 4,4′-diamino-*α*-truxillic acid, and pentafluoropropionic anhydride because the photodimerization of 4-(pentafluoropropionamido)cinnamic acid did not demonstrate dimerization reactivity. From ^1^H NMR spectra, the shape of the chemical shifts of cyclobutane moiety displayed the pattern of *Sin Head-to-tail* dimer, as *α*-type truxillic acid (Figures S3 and S5). In previous work, the stereochemistry of 4,4′-diamino-*α*-truxillic acid was strictly determined by ^1^H NMR and X-ray diffraction [4,5]. In this work, the cyclobutane pattern of the obtained ATA-F1 and ATA-F2 was similar to the previous one from the ^1^H NMR spectrum. Thus, the stereochemistry of the ATA-F1 and ATA-F2 was determined as an *α*-type structure, as shown in Scheme 1. By using these fluorinated bio-based dicarboxylic acids, fluorinated polyamides, PA-F1 and PA-F2 were synthesized in the presence of triphenyl phosphite ((PhO)_3_P), pyridine, and NMP (Scheme 1).

**Figure 1:**
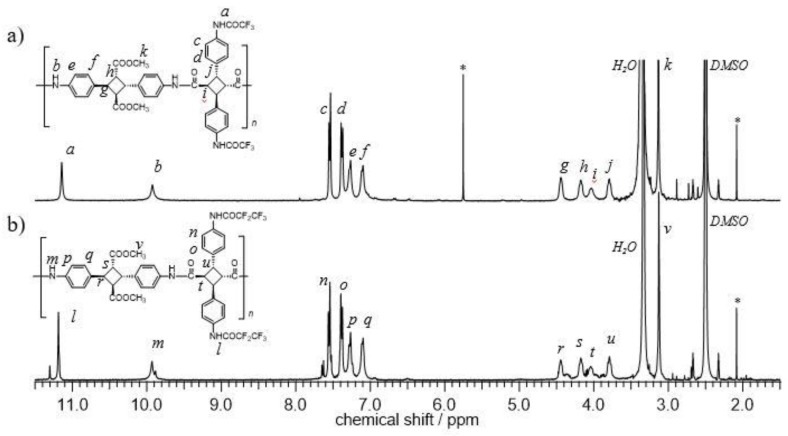
^1^H NMR spectra of the PA-F1 (**a**) and PA-F2 (**b**) (Solvent, DMSO-*d*_6_; [*] indicated residual solvent).

Both polymers of PA-F1 and PA-F2 were quantitatively obtained, and the structure was confirmed by ^1^H NMR measurements (Figure 1). The characteristic peak of the amide proton was observed at 11 ppm (*a*, *l*) and 10 ppm (*b*, *m*), which corresponded to the fluoroalkylated amide and main-chain amide protons, respectively. Furthermore, the peaks of aromatic (*c*–*f* or *n*–*q*) and cyclobutane (*g*–*j* or *r*–*u*) protons were broadened, indicating that polymerization proceeded. There was a difference in the degree of polymerization between PA-F1 and PA-F2, because the solubility of PA-F2 was lower than that of PA-F1, due an increase in the fluorine atoms. In addition, the pentafluoro-group caused the low reactivity of the monomer. This is because the electron withdrawal property improved due to the pentafluoro-group of ATA-F2.

**Figure 2:**
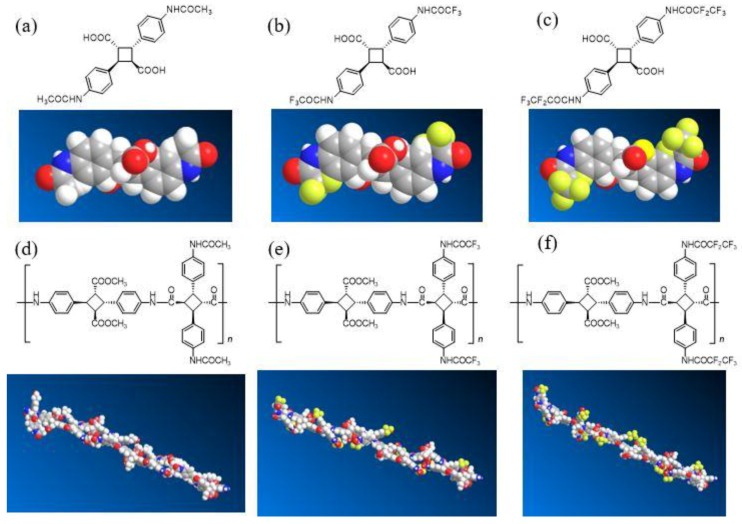
Molecular structure of non-fluorinated and fluorinated dicarboxylic acid (**a**,**b**,**c**) and its polyamides (**d**,**e**,**f**).

The same can be considered with respect to the number-average molecular weight (*M*_n,GPC_) of PA-F2, which was also smaller than that of PA-F1 values (Table 1). Since the molecular weights were determined by GPC, they had a relative molecular weight. The fluorinated polyamide synthesized in this study had fluorine atoms in the side chain, and caused an interaction with the GPC column, which decreased, and the elution time advanced. Therefore, the molecular weight was calculated as being higher than the previous PA. PA-F1 was able to generate a polymer with high molecular weight and high purity. The molecular design of PA-F1 was employed for the subsequent section. The 5% and 10% thermal decomposition temperature (*T*_d5_ and *T*_d10_) of the resulting PA-F1 and PA-F2 were approximately 340 °C and 350 °C, respectively, which were almost same values as the non-fluorinated PAs synthesized in the previous work [5]. *T*_g_ values were approximately 270 °C, and similar to that of the non-fluorinated PA. Based on these results, no change in the thermophysical properties occurred, due to the introduction of the fluoroalkyl group, and a polyamide with high heat resistance could be obtained. The polyamide was dissolved in DMF to prepare a cast film, and the transparency, yellowness index (YI), and contact angle were evaluated. Transparency values were high, at around 90%. The YI of PA-F1 was 3.0, and the film was also colorless. As the side chain became trifluoromethyl and pentafluoroethyl, the colorless and transparency properties increased, while the YI decreased. This was considered to be the result of inhibited packing between the PA molecules from the bulky fluorine side-chain, and the suppression of CT interactions possessed by the PA because of the electron withdrawing effect of fluorine [7]. The contact angle was 80 for PA-F1, and 85 for PA-F2, which was almost the same as the PA without the fluoroalkyl group. These results indicate that the introduction of fluorine to the PA had no effect on the contact angle. This is because the fluoroalkyl group was not closed packed in the PA molecular chain, and thus, there was no increase in the contact angle (Figure 2).

### 3.2. Synthesis of Various Types of Fluorinated Bio-Based Polyamides (PA-F1**a**–**e**)

**Figure 3:**
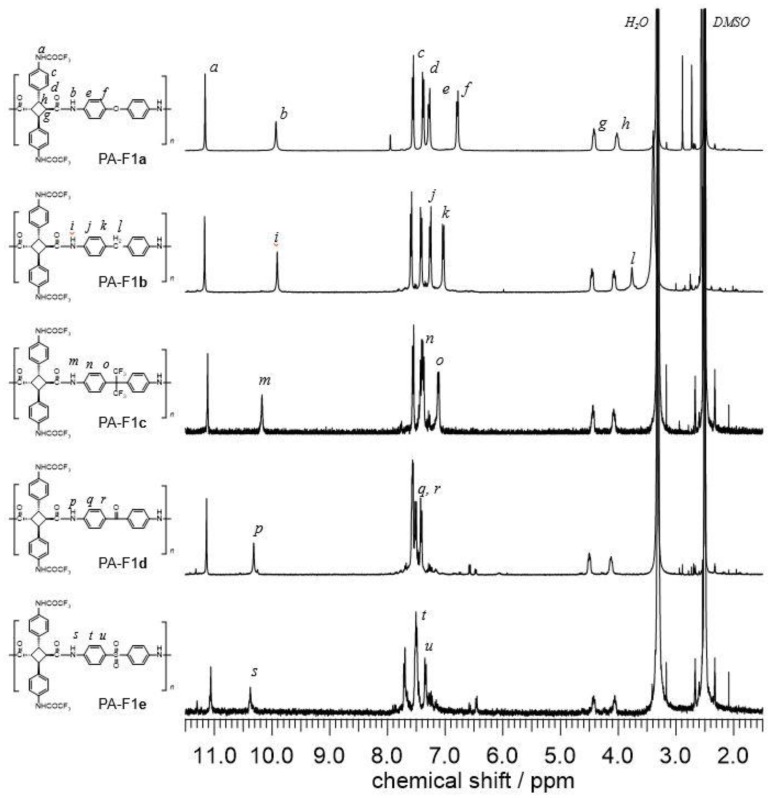
^1^H NMR spectra of the PA-F1**a**, PA-F1**b**, PA-F1**c**, PA-F1**d**, and PA-F1**e** (Solvent, DMSO-*d*_6_).

ATA-F1 and various aromatic diamines, 4,4′-diaminodiphenyl ether (**a**), 4,4′-diaminodiphenylmethane (**b**), 2,2-bis(4-aminophenyl)hexafluoropropane (**c**), 4,4′-diaminobenzophenone (**d**), and 4,4′-diaminodiphenyl sulfone (**e**) were used to synthesize PA-F1**a**–**e** (Table 2). As the results of the ^1^H NMR measurements show, peaks of different chemical shift values appeared between 10.0–10.5 ppm in all PAs (Figure 3). This is attributed to the amide proton of the PAs backbone.

**Scheme 2:**
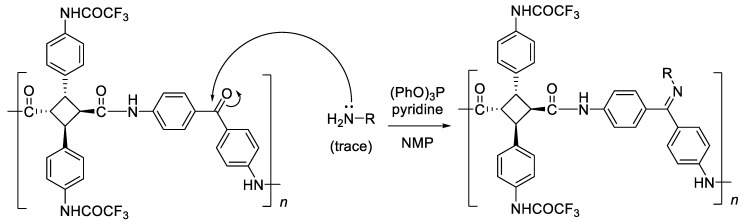
Side-reaction of the synthesis of PA-F1**d** using ATA-F1 and 4,4′-diaminobenzophenone (**d**), in the presence of (PhO)_3_P and pyridine in NMP (“H_2_N-R” means trace amount of residual **d**).

Only PA-F1**a** exhibited a high *M*_n,GPC_ of 1.90 × 10^5^ g mol^−1^. On the other hand, the *M*_n,GPC_ of PA-F1**b**–**e** was 3.17–4.74 × 10^4^ g mol^−1^. The reason that they had a lower molecular weight than PA-F1**a** was because of the difference in the electron-donating properties of the aromatic diamines. The strength of the electron-donating property of the various aromatic diamines, which were used in the polymerization, was **a** > **b** > **c** > **d** > **e**, and the molecular weights also increased in this order. This result indicates that the high electron-donating property increases both the reactivity and the molecular weight of the PAs. Furthermore, with respect to the thermal properties of PA-F1**a**–**e**, all the samples were over 250 °C at *T*_d5_, *T*_d10_, and *T*_g_. The transmittance in the film state was high, and it was 90% or more at 450 nm. In addition, the values of YI were 3.0, 4.4, 4.3, and 3.4 for PA-F1**a**, **b**, **c**, and **e**, respectively, and yellowness was suppressed more than the non-fluorinated PA. PA-F1**d** had a high YI value because imine, which has a strong coloration, was formed by carbonyl reacting with trace amounts of retained amine during polymerization as a side-reaction (Scheme 2). In the contact angle measurement, those with a hydrophobic methylene group (PA-F1**b**) or hexafluoroisopropylidene group (PA-F1**c**) in the diamine moiety had relatively high values, while the hydrophilic carbonyl groups (PA-F1**d**) and the sulfone group (PA-F1**e**) had low values.

## 4. Conclusions

The novel fluorine-containing bio-based PA was synthesized using the fluorinated truxillic acid derivatives synthesized from 4-aminocinnamic acid, ATA-F1, and ATA-F2. In the case of using 4,4′-diamino-*α*-truxillic acid dimethyl ester as a counter monomer of ATA-F1 and ATA-F2, it was found that the colorless and transparency properties improved because of the fluorine side chain. In addition, a slight improvement of water repellency was observed with the introduction of the pentafluoroethyl group, suggesting that larger the fluorine content influences the contact angle. The thermal properties *T*_d5_, *T*_d10_, and *T*_g_, of PA-F1 and PA-F2, demonstrated almost the same values as those of the non-fluorinated PA. This indicates that the introduction of the fluorine group had no effect on these properties. However, transparency could be improved while maintaining a high heat resistance. In the case of PA-F1**a**–**e** using various aromatic diamines as counter monomers of ATA-F1, the reactivity varied depending on the diamine, and it was determined that the electron-donating ability was affected. These fluorinated bio-polyamides exhibit high transparencies and less yellowing, particularly compared to aromatic polymers, and therefore, they can be expected to be used as organic glass, such as poly(methyl methacrylate)s and polycarbonates.

## Figures and Tables

**Table 1:** Molecular weight, thermal, and optical properties of the obtained PA-F1 and PA-F2 *^a^*.

Polymer	*M*_n,GPC_/g mol^−1^*^b^*	*M*_w_/*M*_n_*^b^*	*T*_d5_/°C *^c^*	*T*_d10_/°C *^c^*	*T*_g_/°C *^d^*	Transparency _(450 nm)_/% *^e^*	YI *^e^*	Contact Angle/°
PA-F1	110,000	2.09	341	355	274	88	3.0	80
PA-F2	69,000	2.02	343	358	270	91	3.7	85
PA *^f^*	10,000	1.99	346	361	273	86	4.9	82

*^a^* Reaction conditions: N_2_ atmosphere; temp., 100 °C; time, 4 h; [M]_0_, 1.0 mol L^−1^. *^b^* Determined by DMF/LiBr (0.01 mol L^−1^) GPC using polystyrene standards. *^c^* Determined by TGA. *^d^* Determined by DSC in second heating. *^e^* Determined by UV-Vis with D1925. *^f^* Bio-based polyamide from 4,4′-diamino-α-truxillic acid dimethyl ester and 4,4′-diacetamido-α-truxillic acid [5].

**Table 2:** Molecular weight, thermal, and optical properties of polyamide (PA-F1**a**~**e**).

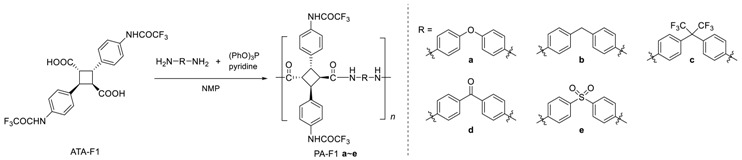								
**Polymer**	***M*_n,GPC_/g mol^−1^*^b^***	***M*_w_/*M*_n_*^b^***	***T*_d5_/°C *^c^***	**T_d10_/°C *^c^***	***T*_g_/°C *^d^***	**Transparency (450 nm)/% *^e^***	**YI *^e^***	**Contact Angle/°**
PA-F1**a**	190,000	1.59	346	359	283	97	3.0	81
PA-F1**b**	61,000	1.53	323	334	255	96	4.4	83
PA-F1**c**	47,400	1.37	314	329	254	98	4.3	82
PA-F1**d**	31,700	1.42	338	359	300	90	9.9	79
PA-F1**e**	34,200	1.37	339	355	263	97	3.4	78

*^a^* Reaction conditions: N_2_ atmosphere; temp., 100 °C; time, 4 h; [M]_0_, 1.0 mol L^−1^. *^b^* Determined by DMF/LiBr (0.01 mol L^−1^) GPC using polystyrene standards. *^c^* Determined by TGA. *^d^* Determined by DSC in second heating. *^e^* Determined by UV-Vis with D1925.

## Supplementary Materials

The following are available online at http://www.mdpi.com/2073-4360/10/12/1311/s1. Figure S1: ^1^H NMR spectrum of 4-(trifluoroacetamido)cinnamic acid, (400 MHz; solvent, DMSO-*d*_6_). Figure S2: ^13^C NMR spectrum of 4-(trifluoroacetamido)cinnamic acid (100 MHz; solvent, DMSO-*d*_6_). Figure S3: ^1^H NMR spectrum of 4,4′-bis(trifluoroacetamido)-*α*-truxillic acid (ATA-F1) (400 MHz; solvent, DMSO-*d*_6_). Figure S4: ^13^C NMR spectrum of ATA-F1 (100 MHz; solvent, DMSO-*d*_6_). Figure S5: ^1^H NMR spectrum of 4,4′-bis(pentafluoropropionamido)-*α*-truxillic acid (ATA-F2) (400 MHz; solvent, DMSO-*d*_6_). Figure S6: ^13^C NMR spectrum of ATA-F2 (100 MHz; solvent, DMSO-*d*_6_). Figure S7: ESI-FT-ICR mass spectrum of the ATA-F1. Figure S8: ESI-FT-ICR mass spectrum of the ATA-F2.
