# Supplementary material for: Fluorinated and Bio-Based Polyamides with High Transparencies and Low Yellowness Index
Source: Polymers (Basel). 2018 Nov 27;10(12):1311. doi: 10.3390/polym10121311 (PMC6401718; doi:10.3390/polym10121311)
Supplement: Supplementary file 1 [file polymers-10-01311-s001.pdf]

Supplementary Materials

Fluorinated and Bio-based Polyamide with High Transparency and Low Yellowness Index

*Kenji Takada [1,2], Yuko Mae [1], and Tatsuo Kaneko [1,2]**

[1] Graduate School of Advanced Science and Technology, Energy and Environment Area, Japan Advanced Institute of Science and Technology, 1-1 Asahidai, Nomi, Ishikawa, 923-1292, Japan; takada@jaist.ac.jp

[2] Japan Science and Technology, JST ALCA, Tokyo 102-0076, Japan

*Correspondence: kaneko@jaist.ac.jp; Tel.: +81-761-51-1631

CONTENTS

- ^{1}H and ^{13}C NMR spectra of 4-(trifluoroacetamido)cinnamic acid, 4,4’-bis(trifluoroacetamido)-*α*-truxillic acid (ATA-F1), and 4,4’-bis(pentafluoropropionamido)-*α*-truxillic acid (ATA-F2)

  ・・・・・・・・・Figure S1−S6

- Mass spectra of ATA-F1 and ATA-F2

  ・・・・・・・・・Figure S7 and S8

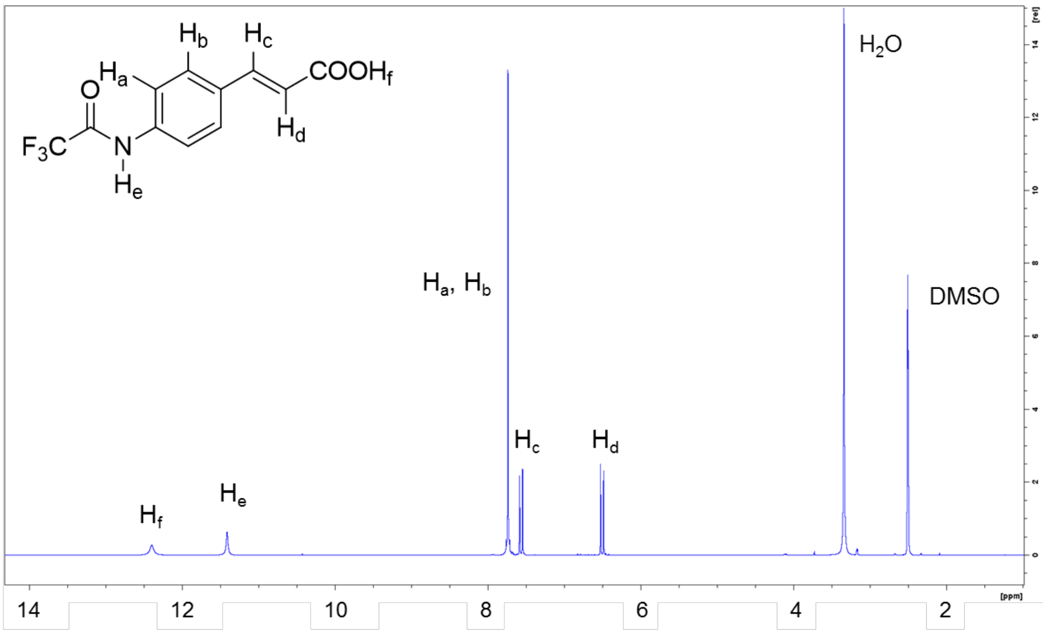

Figure S1. ^{1}H NMR spectrum of 4-(trifluoroacetamido)cinnamic acid, (400 MHz; solvent, DMSO-d_6).

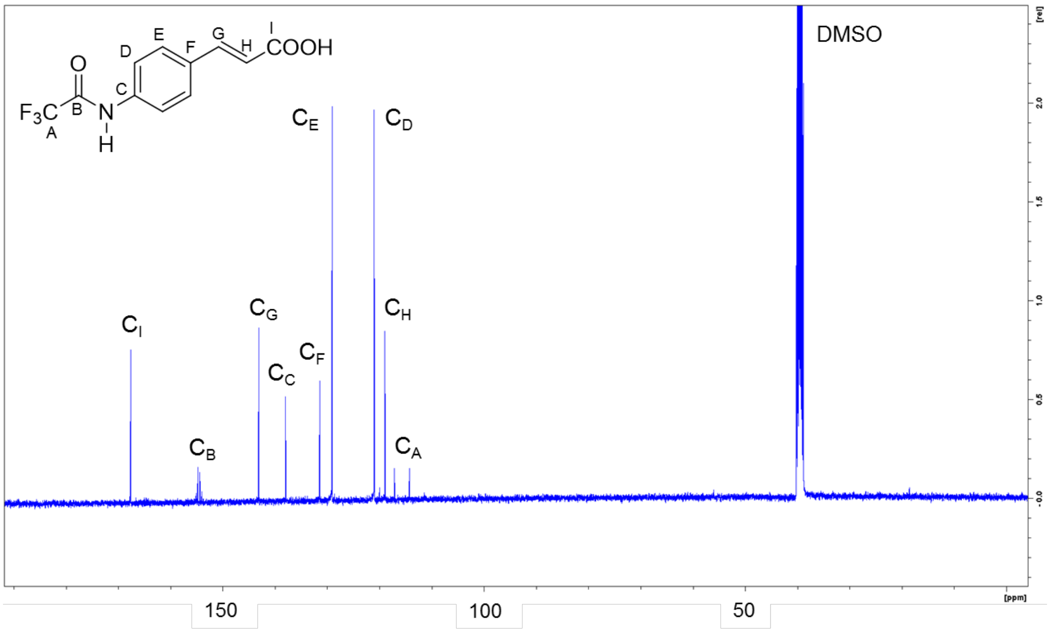

Figure S2. ^{13}C NMR spectrum of 4-(trifluoroacetamido)cinnamic acid (100 MHz; solvent, DMSO-d_6).

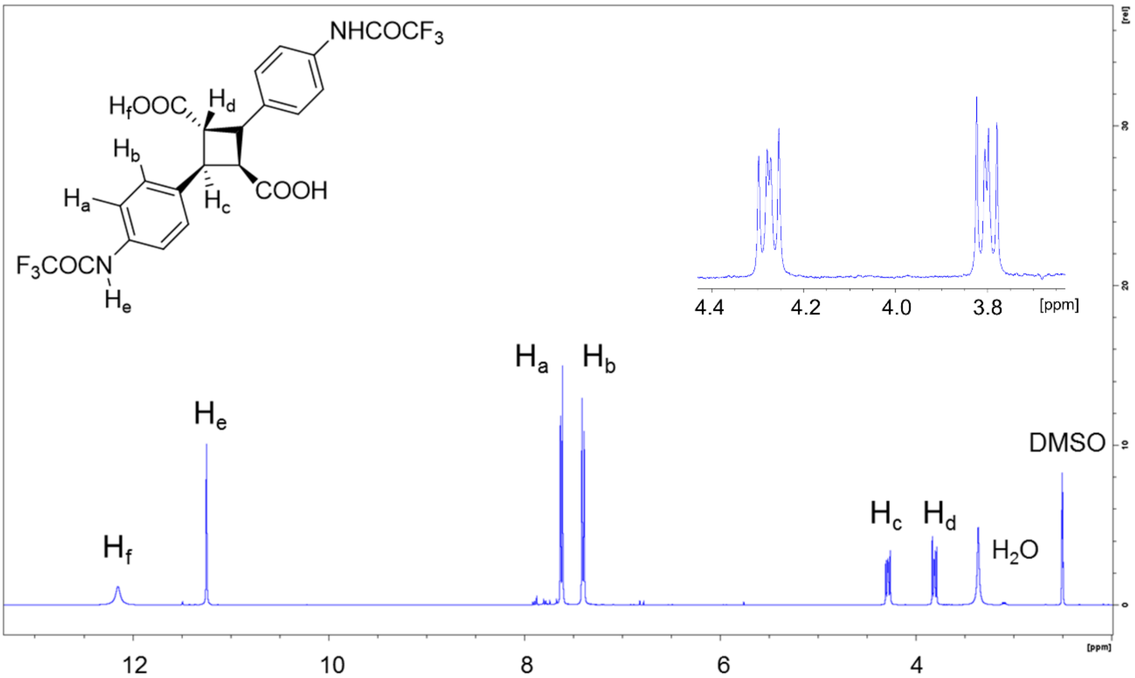

Figure S3. ^{1}H NMR spectrum of 4,4'-bis(trifluoroacetamido)-*α*-truxillic acid (ATA-F1) (400 MHz; solvent, DMSO-*d*$_6$).

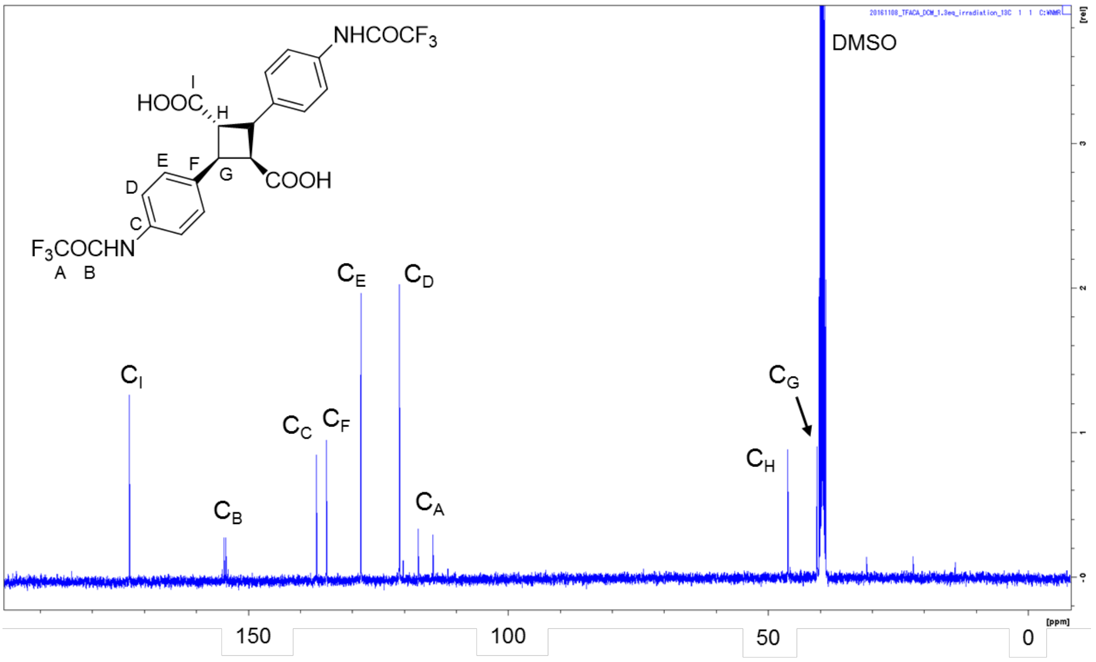

Figure S4. ^{13}C NMR spectrum of ATA-F1 (100 MHz; solvent, DMSO-*d*$_6$).

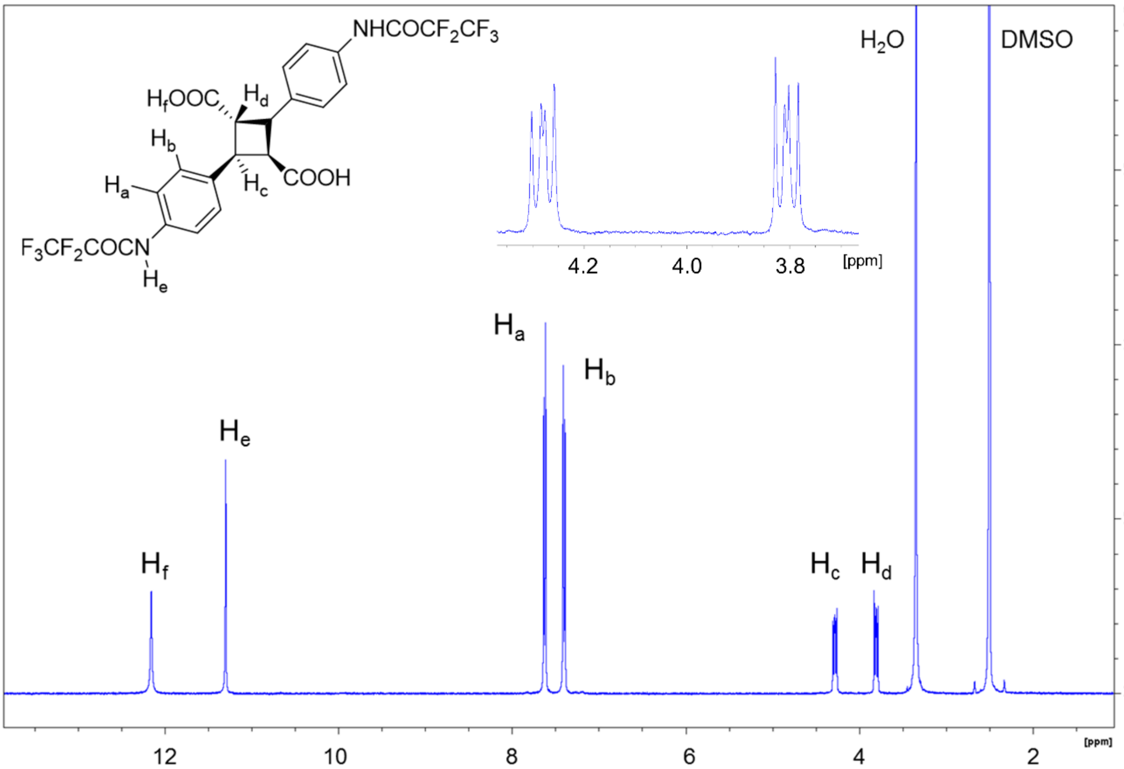

Figure S5. ^{1}H NMR spectrum of 4,4'-bis(pentafluoropropionamido)-α-truxillic acid (ATA-F2) (400 MHz; solvent, DMSO-d_6).

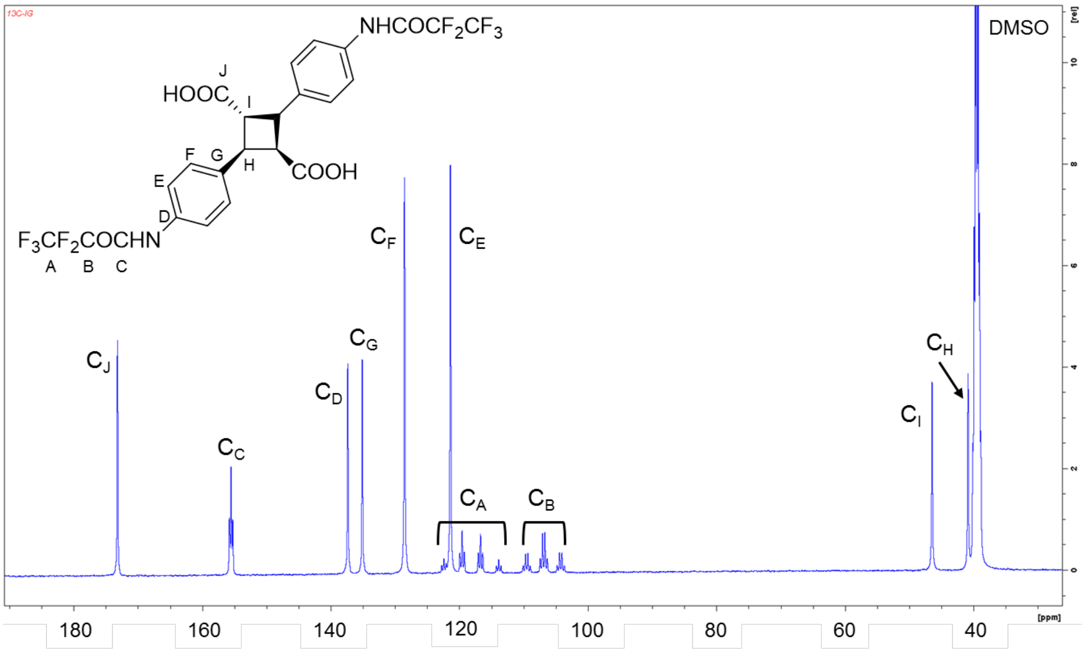

Figure S6. ^{13}C NMR spectrum of ATA-F2 (100 MHz; solvent, DMSO-d_6).

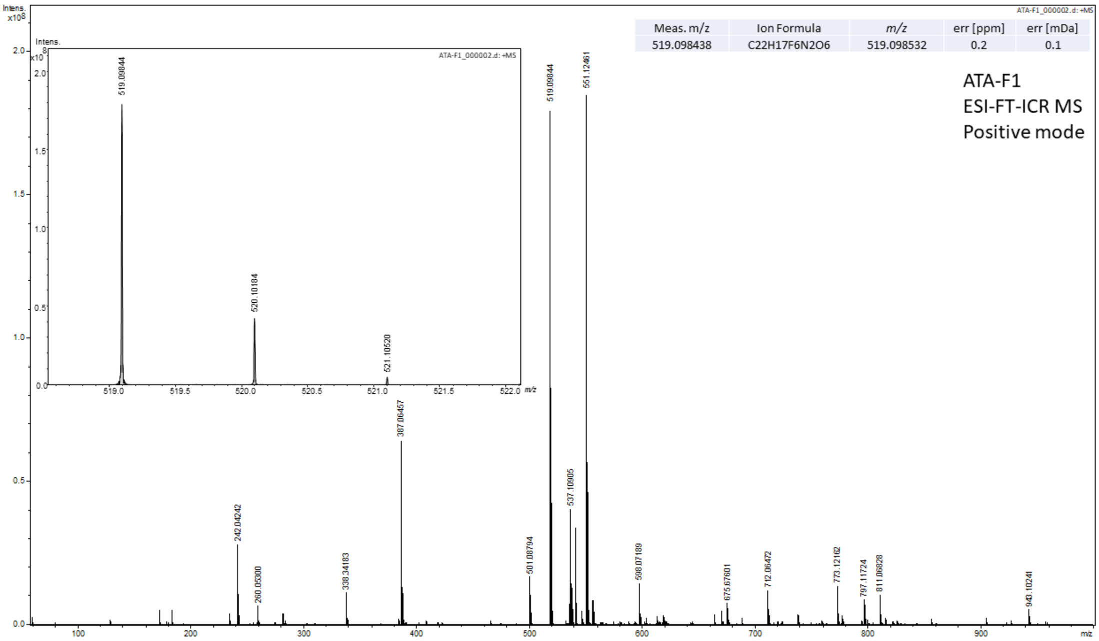

Figure S7. ESI-FT-ICR mass spectrum of the ATA-F1.

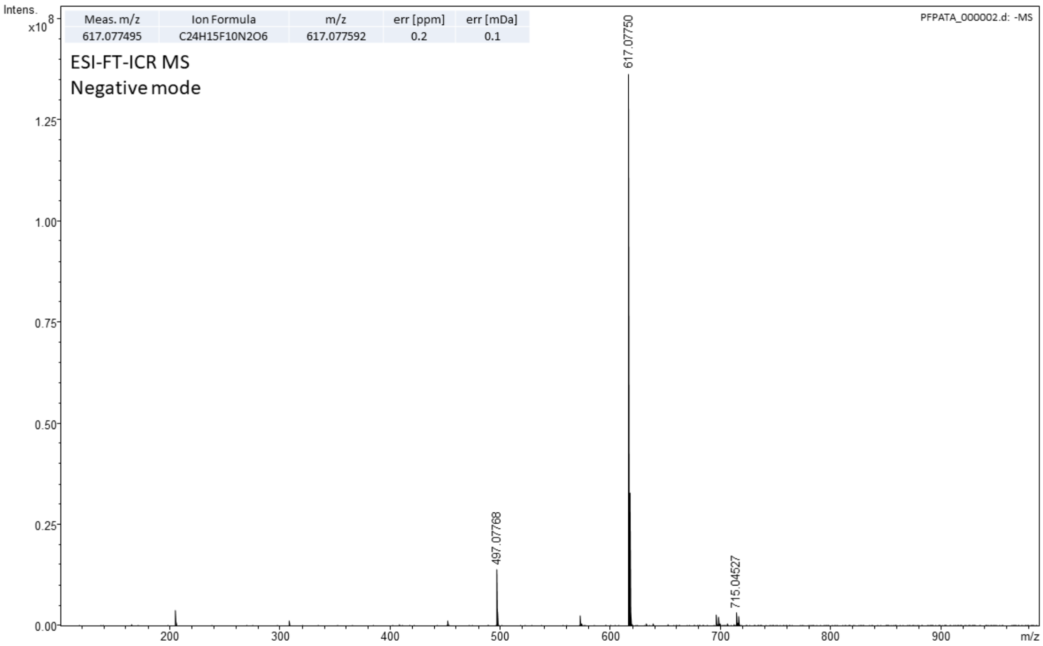

Figure S8. ESI-FT-ICR mass spectrum of the ATA-F2.
